# annot8r: GO, EC and KEGG annotation of EST datasets

**DOI:** 10.1186/1471-2105-9-180

**Published:** 2008-04-09

**Authors:** Ralf Schmid, Mark L Blaxter

**Affiliations:** 1 Department of Biochemistry, University of Leicester, Lancaster Road, Leicester LE1 9HN, UK; 2 The Institute of Evolutionary Biology, University of Edinburgh, King's Buildings, Ashworth Laboratories, West Mains Road, Edinburgh EH9 3JT, UK

## Abstract

**Background:**

The expressed sequence tag (EST) methodology is an attractive option for the generation of sequence data for species for which no completely sequenced genome is available. The annotation and comparative analysis of such datasets poses a formidable challenge for research groups that do not have the bioinformatics infrastructure of major genome sequencing centres. Therefore, there is a need for user-friendly tools to facilitate the annotation of non-model species EST datasets with well-defined ontologies that enable meaningful cross-species comparisons. To address this, we have developed annot8r, a platform for the rapid annotation of EST datasets with GO-terms, EC-numbers and KEGG-pathways.

**Results:**

annot8r automatically downloads all files relevant for the annotation process and generates a reference database that stores UniProt entries, their associated Gene Ontology (GO), Enzyme Commission (EC) and Kyoto Encyclopaedia of Genes and Genomes (KEGG) annotation and additional relevant data. For each of GO, EC and KEGG, annot8r extracts a specific sequence subset from the UniProt dataset based on the information stored in the reference database. These three subsets are then formatted for BLAST searches. The user provides the protein or nucleotide sequences to be annotated and annot8r runs BLAST searches against these three subsets. The BLAST results are parsed and the corresponding annotations retrieved from the reference database. The annotations are saved both as flat files and also in a relational postgreSQL results database to facilitate more advanced searches within the results. annot8r is integrated with the PartiGene suite of EST analysis tools.

**Conclusion:**

annot8r is a tool that assigns GO, EC and KEGG annotations for data sets resulting from EST sequencing projects both rapidly and efficiently. The benefits of an underlying relational database, flexibility and the ease of use of the program make it ideally suited for non-model species EST-sequencing projects.

## Background

Protein sequences from model organisms are generally well annotated. The situation is different for non-model species where often the core of available sequence data comes from expressed sequence tags (ESTs). To date almost one thousand of the species represented in dbEST [1] have at least 100 sequences deposited. Many of these smaller EST sequencing projects are generated by laboratories that do not have the bioinformatics infrastructure available to genome sequencing centers, and there is a need for user-friendly and easy-to-use tools to assist in the functional annotation of sequences for non-model organisms on this scale. Traditionally, annotation for such projects has been based on the descriptor of the best BLAST hit. To go beyond this, tools for the identification of known domains in the sequences of interest, for example InterProScan [2] as a meta-domain search tool, are widely used. For comparative analyses, whether cross-species or cross-libraries, systematic annotation descriptors are very powerful. Gene Ontology (GO) provides a controlled vocabulary to describe gene products [3]. Enzyme commission (EC) numbers are a long-established hierarchical classification scheme for enzymes based on the reaction catalysed [4]. The Kyoto Encyclopedia for Genes and Genomes (KEGG) provides annotation of biochemical pathways for species where the genome has been sequenced [5].

We have developed annot8r, a software tool that facilitates the annotation of new sequences with GO terms, EC numbers and KEGG pathways based on similarity searches against annotated subsets of the EMBL UniProt database [6]. annot8r is a generic tool that can be used for automated annotation of any protein (or nucleotide) sequences, but it has been written predominantly for the annotation of EST datasets. The annotation of EST datasets has some inherent problems, such as redundancy and incompleteness of sequences. We therefore recommend the clustering and the generation of consensus sequences for each cluster before annotation, for example using the PartiGene EST pipeline [7]. Furthermore we would recommend the use of robust peptide translations such as provided by prot4EST [8] to correct error-prone EST datasets for frame shifts. annot8r has been used for the annotation of EST datasets from a wide taxonomic range of organisms including 37 species of free-living and parasitic nematodes in NEMBASE [9,10], the earthworm EST project presented in LumbriBase [11,12], lepidopteran ESTs in ButterflyBase [13,14] and ESTs from the Atlantic halibut [15,16].

## Implementation

### Installation and overview

annot8r has been tested on both LINUX and Mac OS X Darwin platforms. The software is written in Perl and requires a standard Perl installation (5.8.0 or later) and the BioPerl module [17]. On some platforms additional Perl modules may be required (e.g. DBD-Pg) which can be retrieved via CPAN [18]. NCBI-BLAST [19] and postgreSQL (version 7.2 or later, [20]) need to be installed before running the program. To assist the novice user the annot8r user guide includes a list of FAQs covering questions related to the installation of tools annot8r depends on such as NCBI-BLAST, BioPerl and postgreSQL [see Additional file 1]. Once the external tools are installed annot8r is very easy to use via a text-menu driven interface.

**Figure 1:**
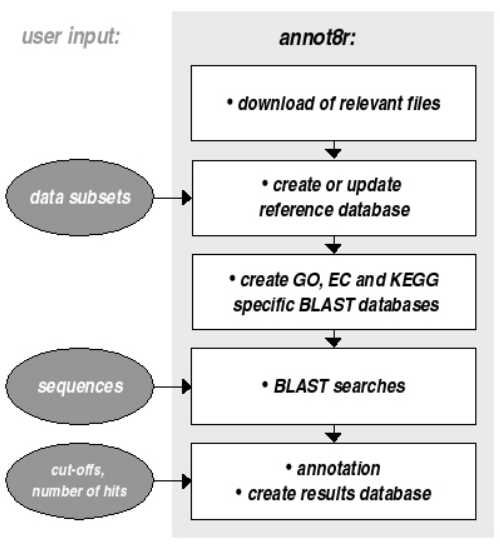
**Overview of annot8r**. The figure describes the data flow for the five main annot8r steps and indicates where, and what type of, user input is required. For the analysis of multiple datasets steps one to three need only be performed once, as they prepare the reference databases for the annotation process. However, we recommend updating the reference databases from time to time to take advantage of the most recent information available.

annot8r is started from a terminal window and takes the user step-by-step through (1) the download of relevant files, (2) the extraction of data from these files, (3) the preparation for BLAST searches, (4) running BLAST searches and (5) the actual annotation (Fig. 1). In principle steps (1), (2) and (3) need only be performed once as they prepare the reference databases for the annotation process. However, we recommend updating the reference databases from time to time, in particular before starting major annotation projects. Protein or nucleotide sequences to be annotated are required, in multi-sequence FASTA text format, as input at step four. The resulting annotations are saved as text files (in comma separated value format) and in a relational database. As we assume that the user of annot8r will be a bench biologist with experience in bioinformatics rather than an experienced bioinformatician, the program comes with an extensive user guide. A step-by-step tutorial covers in detail how to use the program and also gives examples of useful SQL commands to illustrate the power of relational databases for more advanced comparative analyses.

### Retrieving files and generation of the reference database

annot8r offers the user an automated download of the latest versions of all relevant files. All entries of UniProt [6] are downloaded from EMBL in FASTA format and stored as the core protein sequence resource. In addition, files linking GO, EC and KEGG annotations to UniProt identifiers are retrieved. GO annotations for UniProt sequences are provided by the GOA consortium [21]. For GO pie-charts annot8r uses the more general GO-slim terms as defined by the GOA consortium [21]. A list of EC annotations of UniProt sequences is available from the Swiss Institute of Bioinformatics Enzyme project [4]. The KEGG consortium provides a complete set of UniProt proteins that have attached a KEGG orthology category. All information from these files relevant for the annotation process is read into a postgreSQL reference database for efficient look-up. Based on this reference database, annot8r builds three distinct BLAST-searchable subsets of UniProt. Each of these subsets is significantly smaller than the full UniProt resource and contains exclusively entries for which GO, EC or KEGG information is available. This strategy has two major advantages. It reduces the time required for similarity searches compared to a BLAST search against the full UniProt or NCBI nonredundant database. It also ensures that only informative sequences, *i.e*. sequences that can be used to derive GO, EC or KEGG annotation, are present in the database and therefore avoids the risk that informative hits may be lost in a sea of non-informative hits.

### Similarity searches and generation of the results database

**Figure 2:**
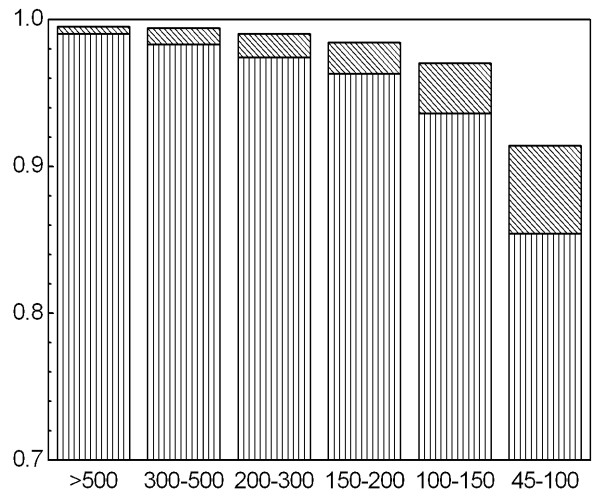
**Performance of annot8r EC-annotation**. The plot shows the fraction of correct EC predictions as a function of BLAST similarity scores. Three categories were defined: all four EC levels correct (*e.g. *EC 1.2.3.4, vertically hatched in figure), the top three EC hierarchy levels correct (*e.g. *EC 1.2.3.x, diagonally hatched) and incorrect. The total number of annotations for each group of BLAST scores is: score >500: 42329; 300–500: 18692; 200–300: 8024; 150–200: 3033; 100–150: 2173; and 45–100: 1459.

To start the BLAST searches against each of these three UniProt subsets the user has to provide the sequences to be annotated as an input file in multi-FASTA format. While we recommend the use of robust peptide translations of EST datasets, annot8r also accepts nucleotide sequences as input (and BLASTX rather than BLASTP is used for searches). The entire annotation process is fully automated, but the user is encouraged to provide input regarding the stringency of the annotation. BLAST score or expect value based cut-offs (see Figure 2 for the impact of cut-off values on EC annotation) are set by the user to define the minimum similarity for hits to be considered. The "number of hits per sequence" defines the maximum number of hits to be considered for annotation. To increase the coverage, but at the risk of an increased number of false positives, we allow, but do not recommend, the inclusion of electronically inferred annotation (IEA) in the GO reference database.

In the analysis step the results of these BLAST searches are parsed and relevant information is retrieved from the reference databases. For each sequence annotation entries that are supported by one or more hits and match the defined cut-offs and limits are collected. For each of these entries (annotation terms) annot8r records the best hit supporting this particular annotation and the corresponding score and e-value for this hit. In addition the number of additional hits also supporting this annotation is recorded. Furthermore, the fraction of hits out of all collected hits for a particular sequence that support this annotation is calculated. This calculation accounts for terms where the maximum number of sequences in the database for a certain annotation is smaller than the number of hits collected, so that in all cases a fraction of 1.0 means maximum possible support.

The annotation results are stored in comma-separated value text files that can easily be read into spreadsheets, and in a relational postgreSQL database. A relational database facilitates more advanced queries, for example the identification of annotation terms which are present in one species, but not in another species, or, annotation terms which are present in all species investigated. Detailed examples illustrating this are given in the tutorial part of the user guide.

## Results and Discussion

### Speed and accuracy of predictions

Removing non-informative entries from the UniProt database and splitting it into three significantly smaller databases specific for GO terms, EC numbers and KEGG pathways before running BLAST searches reduces the time required for the sequence similarity searches compared to a full UniProt search by a factor of ~5. On a single processor 3.6 GHz Intel Pentium workstation the BLAST searches for a set of 1000 typical EST-derived proteins take ~75 minutes against the annot8r databases (GO without IEA, EC and KEGG) as compared to ~400 minutes for the complete UniProt database.

The 'correctness' of annotations based on sequence similarity will depend on factors such as the quality of the annotations in the reference dataset, the specificity of the annotation, whether the sequence belongs to a protein family, and the level of similarity to the reference. This makes estimates of the quality of annotations difficult. To provide the user with some ideas of best-practice cut-offs, we have analysed the relation between sequence similarity and annotation quality for EC annotation. EC annotations have four hierarchy levels. The top level describes the general type of the enzyme reaction. The three sublevels classify the biochemical reaction in ever-greater detail. The UniProt subset containing EC annotations was subjected to a BLAST search against itself. After removing self-hits, the sequences were assigned EC numbers and the annotations sorted according to the underlying BLAST score. Figure 2 shows the fraction of correct EC predictions as a function of BLAST similarity scores. Three categories were defined: all four EC levels being correct, the top three EC hierarchy levels correct, and incorrect. The total number of annotations for each group is given in Figure 1. Even at relatively low BLAST score levels (100–150) annot8r achieves over 96% accuracy in assigning EC annotation to the top three levels.

Collecting not just the first hit, but also a list of top-scoring hits can give rise to alternative or conflicting annotations. We believe that the best strategy in cases such as these is to provide the user with all relevant information necessary to make an informed judgement. Therefore, to assist the user in the assessment of the quality of a particular annotation, annot8r also considers alternative annotations. Based on the e-value or BLAST score cut-off and number of hits that are set by the user, annot8r records for each putative annotation the best hit and its respective scores that suggest this annotation term, the number of additional hits which are also in support of this annotation term, and the fraction of hits better than cut-off supporting each alternative annotation. This allows the user to consider alternative or conflicting annotations and gives guidance as to the distinctness and accuracy of the annotation. For example, if for one particular sequence two EC numbers have a similar score and share the three top EC levels, but display diversity at level four the prediction of the specific substrate used will require a more in-depth analysis while the more general reaction is likely to be correct.

### Comparison with other tools

Other tools are available for the annotation of sequences from non-model organisms with GO terms (for examples see the list provided by the GO-consortium [22]). The most widely used are probably GOtcha [23], which provides quality scored GO-annotation, and InterProScan [2], which along with its annotation of domains and protein motifs also provides high level GO annotation. To our knowledge only a few of these tools annotate with all three ontologies: GO terms, EC numbers and KEGG pathways. BLAST2GO [24] uses NCBI-BLAST via the internet against the NCBI nonredundant protein database and provides GO, EC and KEGG annotation. AutoFACT [25] also offers all three (GO, EC, KEGG) annotations based on BLAST searches against the full UniProt database as part of an annotation package that also covers domain annotation.

The most time consuming step of the annotation procedure is similarity searching. Here annot8r follows a unique route. Instead of searching the full databases (UniProt or NCBI non-redundant) annot8r uses a pre-screening step to generate subsets of UniProt specific to GO, EC and KEGG annotation. The benefit of this is two-fold. As the databases to be searched against are significantly smaller, search times are reduced. We intend to exploit this gain in speed to set up an annot8r web-server in the future. Also, removing non-informative sequences from UniProt before running the BLAST searches avoids the risk of having only non-informative hits in the top hits.

An additional strength of annot8r is the provision of the results in a relational database in addition to flat-files. This enables a skilled user to run more complex search queries on the results. To encourage users with little bioinformatics experience to use this feature, we have given detailed examples in the tutorial part of the user guide [see Additional file 1].

## Conclusion

annot8r is an easy to install and easy to use tool that allows high throughput annotation at low computational cost. It enables the researcher to annotate non-model species sequences with GO, EC and KEGG terms. A relational database makes annot8r particularly suited for comparative studies.

## Availability and requirements

**Project name**: annot8r

**Project home page**: 

**Operating system**: Linux

**Programming language**: Perl

**Other requirements**: BioPerl, CPAN, PostgreSQL, BLAST

**License**: GNU GPL

**Restrictions**: none

## Authors' contributions

RS developed the software and drafted the manuscript. MLB initiated the project and assisted with testing and documenting the software. Both authors contributed to writing the manuscript.

## Supplementary Material

Additional file 1 Zipped tar archive that contains the program, the user guide and sample files needed for running the tutorial. Click here for file
