# Supplementary material for: annot8r: GO, EC and KEGG annotation of EST datasets
Source: BMC Bioinformatics. 2008 Apr 9;9:180. doi: 10.1186/1471-2105-9-180 (PMC2324097; doi:10.1186/1471-2105-9-180)
Supplement: Additional file 1 — Zipped tar archive that contains the program, the user guide and sample files needed for running the tutorial. [file 1471-2105-9-180-S1.gz › annot8r_1.1/annot8r_UG.doc]

**annot8r - a tool for BLAST based GO-EC-KEGG annotation**

**(last updated 02/12/07)**

**Ralf Schmid and Mark Blaxter**

**Department of Biochemistry**

**University of Leicester**

**and**

**The Institute of Evolutionary Biology**

**University of Edinburgh**

R.Schmid@le.ac.uk

nematode.bioinf@ed.ac.uk

**http://www.nematodes.org/bioinformatics**

**Overview**

***annot8r*** has been developed to assist with the annotation of peptide sequences derived from EST datasets using GO-terms, EC-numbers and KEGG biochemical pathways. While ***annot8r*** has been written as part of the Edinburgh EST processing software pipeline, it is a generic tool so that it can be used to annotate any peptide sequences (or nucleotide sequences for that matter). The program is suitable for medium to high throughput data processing and it is freely available for download from <http://www.nematodes.org/bioinformatics/>.

The annotation of GO-terms, EC-numbers and KEGG-pathways in annot8r is based on sequence similarity between the sequences to be annotated and readily annotated protein sequences which are present in the UniProt data resource. For annotation purposes ***annot8r*** creates three separate subsets of UniProt. Each subset contains solely entries which are tagged with GO, EC or KEGG annotation, respectively. The UniProt subsets are formatted as BLAST searchable databases and the sequences to be annotated are then searched against them. In the annotation step the best hits (that is between 1 and 50 hits as set by the user), their associated annotation terms and related information are retrieved and stored in flat files and also, for better query options, in a relational database. The usage of a relational database allows for efficient conditional searches within the annotations, a feature particularly useful for cross-species analysis. A few examples for SQL database search commands are given at the end of the tutorial session in this document.

The UniProt subset created for GO-annotation is based on data provided by the GOA (GO-Annotation@EBI) consortium. ***annot8r*** also prepares data needed for the creation of “GO pie-charts”, this is based on the GO_slim annotations from the GOA project. For EC-annotation ***annot8r*** uses a subset of UniProt based on information provided by the Swiss Institute for Bioinformatics which links UniProt identifiers to EC numbers. The KEGG consortium provides a list of UniProt proteins which have assigned a KO (KEGG orthology) number. This list is used to extract the respective protein sequences from UniProt and to create a KEGG specific subset.

**Requirements**

***annot8r*** runs on Linux/Unix/MacOSX operating systems. It has been thoroughly tested on RedHat, Fedora and debian Linux systems. ***annot8r*** relies on the use of Perl, BioPerl, BLAST and postgreSQL. You have to make sure that the mentioned programs are installed on your system (see FAQs).

**The Software**

***annot8r*** is started by typing “annot8r.pl” in a terminal window. This will load the main menu which lists five available options. These options or sub-menus have to be run in consecutive order. However, ***annot8r*** runs can be interrupted after any submenu. Within every option the user has the choice to run the program for any of GO, EC and KEGG annotation separately or for all of them in one go.

***1. Download relevant files***

Option 1 provides an interface to download all the necessary files for running ***annot8r***. The downloaded files are stored in the directory “downloads”. The unpacked files take up ~6 GB disk space (27/11/2007).

***2. Extract data from files into databases***

In this option all the files downloaded in option 1 are parsed for the relevant information which is exported into three postgreSQL databases for more efficient downstream data processing. The databases “a8r_gobase”, “a8r_ecbase” and “a8r_keggbase” are created, populated and indexed automatically. Before populating “a8r_gobase” the user is asked whether (s)he wants to include GO-terms which are inferred from electronic annotation (IEA) or not. For the annotation of our own datasets we usually do not include IEAs, because we want to avoid false positive annotations as far as possible. If completeness (or “the more the better”) is your main objective using IEAs will give additional annotations.

***3. Prepare your BLAST searches***

Option 3 prepares the relevant databases for the actual BLAST searches. Based on the respective entries in “a8r_gobase”, “a8r_ecbase” and “a8r_keggbase” the corresponding UniProt sequences are extracted and saved as FASTA files in the directory “blast_dbs” under the names “uniprot_GO.fsa”, “uniprot_EC.fsa” and “uniprot_KEGG.fsa”. After sequence extraction the three FASTA files are BLAST formatted to create the databases necessary for the similarity searches.

***4. Blast your sequences***

Option 4 will ask you for a file containing the sequences to be annotated. A single file holding all the input protein or nucleotide sequences in FASTA format (as for example provided by prot4EST) is required as input file for the BLAST search. The user will be asked for name and location of this file. If the file is found a BLAST run is started. As BLAST searches can be very time consuming, this step can also be performed outside of the main program, for example using a 'BLASTfarm'. The results of the BLAST searches are stored in the directory “blast_out”.

***5. Annotate your sequences***

In option 5 each BLAST hit for every query sequence is parsed to extract relevant information from the BLAST output and relate it to the GO, EC and KEGG information processed in option 2. For each query this information is either exported into an existing postgreSQL database (this could for example be a database created by PartiGene) or alternatively a new database is created. To handle the data processing the user needs to set an e-value or bit score cutoff for the sequence similarity required to accept an annotation. Setting a meaningful cutoff is a tricky task. Setting it too stringent may result in missing out on potentially interesting annotations, however setting it too lenient will increase the number of wrong annotations. In general, electronically inferred annotations as the ones provided by ***annot8r*** should be taken with some caution.

***6. The database tables***

***annot8r*** creates the three database tables “a8r_blastgo”, “a8r_blastec” and “a8r_blastkegg” to hold the annotation entries. Each of these tables has a column “pept_id” which stores the identifier of the annotated protein. The columns “besthit” (the hit on which an annotation is based), “bestscore” and “bestev” (the respective BLAST bit score and e-value for this hit), “hitnum” (the number of hits better than the cut-off supporting this particular annotation), “maxhit” (the number of entries in the database for this annotation, this is the number of possible hits that could support this annotation) and “fraction” (“hitnum” divided by “maxhit”, if the number of hits collected is smaller than “maxhit” the smaller number will be used instead of “maxhit”). Please see tutorial for an example.

In addition the table a8r_blastgo has the entries “go_term” which holds the number of the GO-term, “PCF” which gives away the aspect (biological process, molecular function or cellular component) this GO-term belongs to, “descr” - a description of the respective GO-term and “slim” - the corresponding higher level GO-term which is useful for pie-chart type analysis of GO-term distribution. The table “a8r_blastec” has the additional entries “ec_id” for EC number and “descr” for the description of it. In addition to the entries common to all three tables “a8r_blastkegg” has entries for “ko_id” - the KEGG orthology identifier on which the annotation is based, “path” - the corresponding biochemical pathway to the KEGG orthology identifier, and “descr” - a short description of the pathway.

**A short tutorial**

***How to run annot8r - an example***

In the following tutorial any user input is printed in **courier font**. We will use the file test.fsa which is provided with the ***annot8r*** package as input sequences.

The program is started by opening a terminal window and typing **annot8r.pl**. This will open the main menu and offer you five options (and an option to exit the program) to proceed.

1. Select the “Download relevant files” option **1**. This opens the “Downloads” menu, enter **4** for “Download everything”. During the download process on-screen information about the progress being made is continuously updated. All the downloaded files are saved in the directory “downloads”.
2. After the download process is finished, annot8r takes you back to the main menu. Next, enter **2** to select the option “Extract data from files into databases”. Choose **4** “Create or update all of them” to prepare all the relevant databases in one go. Now all the files downloaded in option one are parsed, the relevant data extracted and stored in three databases “a8r_gobase”, “a8r_ecbase” and “a8r_keggbase”. Answer **n** to exclude GO-terms inferred from electronic annotation for the reference database “a8r_gobase” as this will make the tutorial run much faster. Nevertheless, this step will take some time, annot8r keeps you updated about the numbers of entries processed so far. At the time of writing this tutorial there are ~350000 (~11000000 if including IEA) entries for GO, ~80000 for EC and ~400000 for KEGG to process. At the end of the data extraction you will be redirected to the main page.
3. From the main page select option **3** “Prepare your Blast searches” to create BLAST searchable databases for the three UniProt subsets. Choose **4** “Create all of them”. At the day of writing this tutorial the process creates three BLAST-formatted databases uniprot_GO.fsa, uniprot_EC.fsa and uniprot_KEGG.fsa comprising 73000 (2200000 if including IEA in step 2), 75000 and 375000 sequences, respectively.
4. From the main page select option **4** “Blast your sequences”. Within this option select **4** to choose “Run all of them”. Next you are asked for a sequence input file. This input file should contain the sequences of interest in FASTA format. To use the test file provided with the annot8r package type “**test.fsa**” (make sure that this file is in your working directory). As this file holds protein sequences select answer **1** to the question ”Does test.fsa contain protein or nucleotide sequences?”. Now the BLAST similarity searches start. BLAST searches can take a substantial amount of time. After the BLAST searches are finished you are redirected to the main page. The results are stored in the directory “blast_out”. For the input file “test.fsa” three output files “test.fsa_GO.out”, “test.fsa_EC.out” and “test.fsa_KEGG.out” are created. If you have access to a fast BLAST service such as a “BLAST farm” you also can run your BLAST searches outwith annot8r. Simply place the output of the external BLAST searches (the files have to end with “GO.out”, “EC.out” and “KEGG.out”) in the directory “blast_out”.
5. To start the annotation process select option **5** “Annotate your sequences”. From the “Annotation” menu choose **4** “Annotate all of the above” for obtaining GO, EC and KEGG annotation in one go. After this annot8r checks whether the BLAST results required for annotation are available and if they are it asks the user for the name of the database to store the annotations. Enter **a8r_test** as database name for the tutorial run. annot8r checks whether this database is available on the system. If not you will be asked whether annot8r should proceed and create it. Answer **y** to do so. Next, you have to decide what cut-off to use for accepting annotation terms based on sequence similarity. annot8r offers two alternative ways for that. The first is based on BLAST bit-scores, the second on BLAST expect values (e-values). Bit-scores do not depend on the size of the BLAST database used while e-values do. (see BLAST manual for details on this). For our example select **2** to use a bit-score cut-off. This is followed by some information regarding meaningful cutoffs, please read carefully. Finally you are asked to enter a bit-score cutoff. Enter **75** for our example. When you are asked for the number of BLAST hits for each sequence to be databased, enter **10** for our test run. Now the BLAST output is parsed, the relevant information extracted and stored in the database a8r_test. In addition the information is saved in a series of comma separated flatfiles (.csv) which are stored in the directory “output”. Also in this directory the data necessary to create GO pie-charts based on GO-slim annotation are saved as “piedata_C”, “piedata_F” and “piedata_P”. After the program is finished go back to the terminal by answering **n** to the question whether you do want to continue.
6. To access the database from a terminal window type ***psql a8r_test***. This opens a command line postgreSQL interface. In the following a few commands are demonstrated to illustrate the power of relational databases. To list the available database tables a8r_blastgo, a8r_blastec and a8r_blastkegg type ***/d***. The command ***/d a8r_blastgo*** shows the column headers of the a8r_blastgo table. Type ***select * from a8r_ blastgo;*** (don't forget the semicolon), this command will list all the available GO annotation in the database. To select all proteins which are annotated with GO-term 0005515 based on a hit with an e-value better than 1e-30 you would use: ***select pept_id from a8r_blastgo where go_term = 'GO:0005515' and bestev < '1e-30';*** . To retrieve the complete annotation for the KEGG pathway 00010 just enter ***select * from a8r_blastkegg where path = '00010';***. The command ***select ko_id, path, descr from a8r_blastkegg where ko_id in (select ko_id from a8r_blastkegg where pept_id ~ 'ZPP') and pept_id ~ 'NBP' order by path;*** will retrieve KEGG orthology terms, pathways and pathway descriptions for all occasions where a certain KEGG orthology term is present in both, sequences starting with NBP and ZPP, and order them by pathways. In the example given the first three letters of the sequence identifiers stand for the species *Nippostrongyloides brasiliensis* and *Zeldia punctata*. A carefully chosen system of sequence identifiers is very powerful for cross species analysis. Another example for a species specific search is ***select distinct ec_id from a8r_blastec where ec_id not in (select ec_id from a8r_blastec where pept_id ~ 'ZPP') and pept_id ~ 'NBP';*** which retrieves EC-numbers that have been used in the annotation for *Nippostrongyloides brasiliensis* but not for *Zeldia punctata*.
7. To assist the user with the judgement of the reliability of a certain annotation annot8r provides a condensed summary of the underlying BLAST searches. We will explain the meaning of each column in the following two examples. For the first example use: ***select * from a8r_blastec where pept_id = 'NBP00285_1';*** this gives the following result on from test dataset:

| pept_id | ec_id | descr | besthit | best-score | bestev | hit-  num | maxhits | frac-tion |
| --- | --- | --- | --- | --- | --- | --- | --- | --- |
| NBP00285_1 | 1.1.1.27 | L-lactate dehydrogenase | Q27888 | 198 | 2e-51 | 10 | 233 | 1 |

annot8r suggests that NBP00285_1 is a putative L-lactate dehydrogenase (EC 1.1.1.27). This annotation is derived by BLAST similarity of NBP00285_1 to Q27888, which is annotated as EC 1.1.1.27 . The respective BLAST similarity score is 198 which corresponds to an e-value of 2e-51. 233 sequences with the annotation EC 1.1.1.27 are in the EC database. Ten hits have been collected with a score better than the cutoff value (remember that both, the maximum number of hits collected and cutoff to be used, are set by the user). As all of these ten hits have the annotation EC 1.1.1.27 , therefore the fraction of hits supporting the notion that NBP00285_1 is a putative L-lactate dehydrogenase is “1”.

The second example covers a case of ambiguous annotation. The SQL command ***select * from a8r_blastec where pept_id = 'ZPP00164_1';*** retrieves two potential annotations for ZPP00164_1.

| pept_id | ec_id | descr | besthit | best-score | bestev | hit-  num | maxhits | frac-tion |
| --- | --- | --- | --- | --- | --- | --- | --- | --- |
| ZPP00164_1 | 5.1.3.2 | UDP-glucose 4-epimerase | Q8R059 | 135 | 5e-32 | 10 | 46 | 1 |
| ZPP00164_1 | 5.1.3.3 | Aldose 1-epimerase | P04397 | 111 | 8e-25 | 3 | 17 | 0.3 |

The best ten hits (the maximum number collected) all point to EC 5.1.3.2 with Q8R059 being the hit with the best score. Therefore, the fraction of hits supporting the EC 5.1.3.2 annotation equals “1”. But there is an alternative annotation to be considered, EC 5.1.3.3 . This annotation is supported by three of the top ten hits (fraction = 0.3). Both annotations are reported in the annot8r output. For this example it is worth to point out that the three hits supporting the EC 5.1.3.3 annotation also support the EC 5.1.3.2 annotation (hence the fraction off 1.0 for EC 5.1.3.2.) as e.g. P04397 is one of the annotated sequences in the EC database which has more than one EC number (EC 5.1.3.2 and EC 5.1.3.3).

8. Type ***\q*** to exit the postgreSQL interface.

For further questions, comments, problems or bug reports please contact [nematode.bioinf@ed.ac.uk](mailto:nematode.bioinf@ed.ac.uk)

**FAQs**

**1 How do I unpack the annot8r.tar.gz file ?**

In a terminal window type **gunzip annot8r.tar.gz**. Next, to untar the unpacked file type **tar xvf annot8r.tar**. This will create a new directory “annot8r” and save the following files in this directory: annot8r.pl, README, test.fsa and annot8r.pdf.

**2 What is my path?**

The user's path is a variable defining a set of directories where the OS looks for executable programs. It is recommended to copy the file annot8r.pl into one of these directories, for example /usr/local/bin. The way to set or modify your PATH variable depends on the shell you are using, therefore you first need to find out what shell you are using. Simply type: **echo $SHELL**. If you are using a c-type shell (csh, tcsh) open your ~/.cshrc file and modify (or add) the following line to include the directory /usr/local/bin: **setenv PATH “/usr/software/exec/:/usr/local/bin”**. Alternatively, for a bash-type shell (bash, sh) open ~/.bashrc in a text editor and modify or add the PATH line accordingly: **export PATH=$PATH:/usr/software/exec:/usr/local/bin;**. To make the change active you need to “source” the respective file by running **source ~/.cshrc** or **source ~/.bashrc** respectively. Please note, it is important that the “export PATH” and “setenv PATH” entries in ~/.bashrc and ~/.cshrc are in a single line.

**3 How do I install BioPerl ?**

There are different ways to do this, but the one suggested here should also work for essentially any other Perl module. First download the package from [www.BioPerl.org](http://www.bioperl.org/) and unpack the downloaded file as described in FAQ 1. Then change your working directory to the directory “BioPerl-1.2” created by the tar command by typing **cd BioPerl-1.2**. Have a quick look at the README and INSTALL files for details of the module and installation (in some cases there will be only one of these files). Then, unless the instructions in README or INSTALL tell you differently, issue the following commands: **perl Makefile.PL** followed by **make** and **make test**. It is often not a problem if a few (sub)-tests fail. After the tests login as root (or use the “su” [Linux/UNIX] or “sudo” [OSX Darwin] command) and run the command for placing the module in the correct place: **make install**. Now everything should be in place.

**4 How do I install BLAST ?**

Go to NCBI BLAST download webpage <http://www.ncbi.nlm.nih.gov/BLAST/download.shtml> and download the precompiled “blast” suite for your platform (for example, this is linux-ia32 for standard Linux installs). Unpack the file blast-2.2.13-ia32-linux.tar.gz (version numbers may be different) and copy the executables into the /usr/local/bin directory: **cp blast-2.2.13/bin/* /usr/local/bin**. After this move the install directories to /usr/local/software with the command **mv blast-2.2.13/ /usr/local/software**. BLAST requires two environmental variables to be set. In our example BLASTMAT needs to point to /usr/local/software/blast-2.2.13 where the BLAST matrices have been installed. BLASTDB points to the directory where you will store BLAST databases. For example this could be /home/db/blastdb. See FAQ5 for how to set an environmental variable.

**5 How do I set environmental variables ?**

An example for an environmental variable is PATH (see FAQ 2) so setting these can be done analogous to PATH. To set the variable BLASTMAT and assuming you are running a bash shell open the file ~/.bashrc and add a line **export BLASTMAT=/usr/local/software/blast-2.2.13;**.

**6 How do I find out whether postgreSQL is already installed?**

For most setups the command **locate psql** should find the postgreSQL executable (if present on the system). You would expect to find it in a directory were usually executables are kept such as /usr/bin/psql. If your system uses rpm installs you can check for these with the command **rpm -qa | grep -i postgres**

**7 How do I install postgreSQL ?**

Download, unzip and untar the latest version of postgreSQL (available from [www.postgresql.org](http://www.postgresql.org/)), detailed information on postgreSQL and the installation process can be found there as well. First you need to run the configuration script (you might want to modify some of the settings in this script to accommodate your needs) **./configure**. Then, as described previously for the BioPerl installation, run the **make** command. After this log in as superuser (root) and run the **make install** command. Also as superuser create an user account for user postgres (**adduser postgres**). Create a new directory for postgreSQL databases to be stored with the command **mkdir /usr/local/pgsql/data**. To give the ownership of this directory to the user postgres run **chown postgres /usr/local/pgsql/data**. Then login as user postgres (or as superuser you can use the **su postgres** command). As user postgres you have to initialise postgreSQL with the command **/usr/local/pgsql/bin/initdb -D /usr/local/pgsql/data/** . Now the installation is complete. To start the program use the command (still as user postgres) **usr/local/pgsql/bin/postmaster -D /usr/local/pgsql/data >logfile 2>&1**. To create a test database run **/usr/local/pgsql/bin/createdb test**, to access the test database type **/usr/local/pgsql/bin/psql test**. It is also advisable to create postgreSQL user accounts for individual users of annot8r, as this will allow them to create their own databases. See FAQ 10 for the respective commamd.

**8 How do I start postgreSQL ?**

Login as user postgres and follow the instructions at the end of FAQ 7.

**9 How do I find out whether postgreSQL is running already ?**

**ps -ef | grep postmaster** should list any postmaster processes (i.e. postgreSQL) running.

**10 How do I create a postgreSQL user account ?**

Iogging in as user postgres and entering the command **createuser user1** will create a postgresql user account for user1.

**11 How can I get rid of the longish sermon about setting a cut-off ?**

Use a text editor to open the annot8r.pl script. In line 37 set the $quiet variable to 1 (see comments in the script).

**12 How reliable are the annotations ?**

Meaningful benchmarking for automated annotation is a far cry from trivial. We have attempted some benchmarking for the EC annotation. The annotation of all sequences in uniprot_EC.fsa based on BLAST searches against itself (while ignoring self-hits) gives the following numbers:

| Score for hit | % correct annotations for EC X.X.X.X | % correct annotations for EC X.X.X.- | Total number of annotations |
| --- | --- | --- | --- |
| >500 | 99.0 | 99.5 | 42329 |
| 300-500 | 98.3 | 99.4 | 18692 |
| 200-300 | 97.4 | 99.0 | 8024 |
| 150-200 | 96.3 | 98.4 | 3033 |
| 100-150 | 93.6 | 97.0 | 2173 |
| 40-100 | 85.4 | 91.4 | 1459 |
| <40 | n.a. | n.a. | 235 |

For example out of the 2173 annotations which were based on a score for the best BLAST hit between 100 and 150 93.6% had all four levels of the EC terms correct, an additional 3.4% had the top three levels correct and the remaining 3.0% less than that.

But of course this dataset probably does not represent the dataset you will be using. Use your common sense as you do when analyzing BLAST results.

**13 What cut-offs should I use ?**

It depends … (and see FAQ 12)

**14 I am pleased to see that I can get rid of IEA based GO-terms in the reference database, but I also don’t trust 2-hybrid data and would like to exclude them as well. How can I achieve this?**

You need to hack the script, but it is very easy. Have a look at lines 40 to 60 of annot8r.pl where the more stringent data set is defined.

**15 Why is the download option not working properly?**

Assuming your computer is connected to the internet the most likely explanation is that one or more of the files annot8r tries to download has been moved or is in the process of being updated. Open a web browser and see what happens if you copy the respective URL from the annot8r.pl script. You may need to modify the web address in the script, please inform the authors via nematode.bioinf@ed.ac.uk so that this can be fixed in the script.

**16 What should I do if my question is not covered in this FAQ ?**

Contact nematode.bioinf@ed.ac.uk, but keep in mind that the people writing and maintaining this software are scientists as you are and very busy. So please try to sort out any Linux, BLAST or postgreSQL related problems with the help of your local gurus, system administrators, computing support or Google. When reporting a problem a simple statement such as “annot8r doesn't work” is not particularly useful, please provide some details and also try reproduce the problem. If you can't reproduce it, it will be even more difficult for us to act upon it.
